# Examining the expression of low-density lipoprotein receptor (*LDLR*) and low-density lipoprotein receptor-related protein 6 (*LRP6*) genes in breast cancer cell lines

**DOI:** 10.22099/MBRC.2024.48583.1882

**Published:** 2024

**Authors:** Hamid Behrouj, Mehran Erfani, Pooneh Mokarram

**Affiliations:** 1 Department of Biochemistry, School of Medicine, Shiraz University of Medical Sciences, Shiraz, Iran; 2 Behbahan Faculty of Medical Sciences, Behbahan, Iran; 3 Department of Biochemistry, School of Medicine Hormozgan University of Medical Sciences, Hormozgan, Iran; 4 Autophagy Research Center, Department of Biochemistry, Shiraz University of Medical Sciences, Shiraz, Iran

**Keywords:** Breast cancer, Cholesterol, Wnt/β-catenin signaling, LDLR, LRP6

## Abstract

Cholesterol and the Wnt/β-catenin pathway have an effective role in the proliferation, survival, drug resistance, immune exhaustion, and metastasis of all types of cancer cells. Considering the role of LDLR and LRP6 proteins in cholesterol uptake by cells and activation of Wnt/β-catenin pathway, this study aims to examine the gene expression of *LDLR* and *LRP6* in cell lines of breast cancer. Human breast cancer cell lines MCF7, MD468 and SKBR3 were cultured in suitable conditions and after extracting total RNA from them, real-Time PCR was used to measure the levels of gene expression for *LDLR* and *LRP6*. Our results showed that the expression of *LDLR* and *LRP6* genes is significantly increased in MCF7 and MD468 cells compared to SKBR3 cells. These results suggest that *LRP6* and *LDL*R can be considered as a therapeutic target in tumors that have a genetic profile similar to MCF7 and MD468 cells.

## INTRODUCTION

Breast cancer is a frequently diagnosed cancer in women and is the second leading cause of cancer-related deaths. Breast cancer can be categorized into three primary types: hormone receptor-positive (ER+/PR+), human epidermal growth factor receptor-2 overexpression (HER2+), and triple-negative (TNBC) [1]. There are a lot of diverse cellular signaling in the initiation and progression of breast cancer which restrict the efficacy of current therapies.

Obesity and hypercholesterolemia are breast cancer risk factors that have a negative effect on the effectiveness of treatment [2, 3]. Higher amounts of low-density lipoprotein (LDL), plasma cholesterol, triglycerides, and lower levels of high-density lipoprotein (HDL) has been reported in breast cancer patients [4]. An intriguing discovery was made that implicates LDL in the susceptibility of breast cancer cells to radiotherapy [5]. Diverse differentiation incidents are mainly affected by the Wnt/β-catenin pathway which could lead to tumor initiation if abnormally activated. After the Wnt ligands attach to their receptors on the cell surface, which are the frizzled (Fz) family receptors and the low-density lipoprotein receptor-related proteins 5 and 6 (LRP5 and LRP6), the transcription factor β-catenin becomes stable and migrates to the nucleus [6]. There, it controls the expression of genes. Besides, a growing number of studies have shown that LRP6 is increased in various types of cancers and leads to tumor progression [7, 8]. Given the significance of LDLR and LRP6 in facilitating cholesterol entry into cells and activating the Wnt/β-catenin pathway, which ultimately contributes to the maintenance and proliferation of cancerous cells, this study aims to examine the gene expression of *LDLR* and *LRP6* in breast cancer cell lines.

## MATERIALS AND METHODS

**Cell culture: **The current research involved acquiring MCF7, MD468, and SKBR3 human breast cancer cell lines from the National Cell Bank of Iran (located in Tehran) and growing them in RPMI-1640 medium containing 10% fetal bovine serum and 1% penicillin/streptomycin at 37°C in a humid environment with 5% CO_2_.

**Real-Time PCR analysis: **The total RNA was extracted from the cultured cells using the BIOZOL-RNA extraction reagent. Total RNA was converted to single-stranded cDNA by utilizing the Fermentase cDNA Synthesis kit. The SYBR Green and 7500 real-time PCR system were utilized to conduct the real-time polymerase chain reaction. The 2^-ΔΔCt^ formula was used to calculate and normalize the relative mRNA values of *LDLR* and *LRP6* genes, with the glyceraldehyde 3-phosphate dehydrogenase internal control. Table 1 shows the primer pairs used for quantitative PCR.

**Table 1:** The primers sequence used for real-time PCR

**Genes **	**Forward primer**	**Reverse primer**
*GAPDH*	5′-CGACCACTTTGTCAAGCTCA-3′	5′-AGGGGTCTACATGGCAACTG-3′
*LDLR*	5′ - GAACCCATCAAAGAGTGCG- 3′	5′ - TCTTCCTGACCTCGTGCC-3′
*LRP6*	5′ -CACTTACTTCCCTGCAATTTTGAACC3′	5′ - TGGCCTGTAGCTGTATGACCTATG-3′

**Statistical analysis: **The GraphPad Prism version 6 software was utilized to determine the statistical significance of the tests. To make the comparisons, one-way analysis of varicance (ANOVA) was conducted, followed by Dunnett's test, with statistical significance set at P<0.05.

## RESULTS AND DISCUSSION

**Figure 1:**
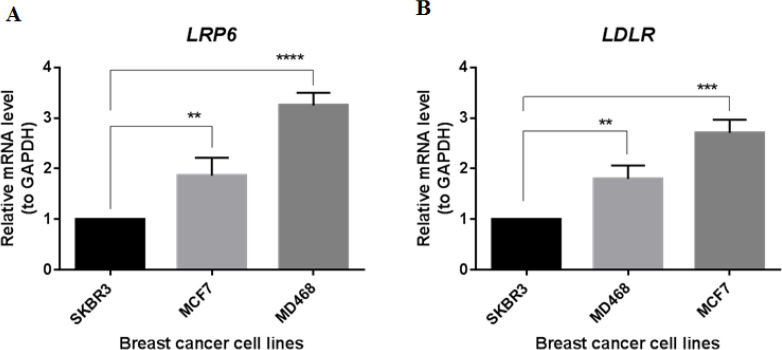
Baseline *LRP6* (A) and *LDLR* (B) genes expression in breast cancer cell lines. Real-time polymerase chain reaction was utilized to measure the expression of the *LRP6* and *LDLR* genes in the specified cell lines. To make the comparisons, one-way ANOVA was conducted, followed by Dunnett's test, with statistical significance set at P<0.01 for ** and P<0.0001 for ****.

As shown in Figure 1, MCF7 and MD468 cells express the *LRP6* and *LDLR* genes significantly more than SKBR3 cells. Similarly, our results also showed that the *LDLR* gene in MCF7 and MD468 cells has a significant increase in expression compared to SKBR3 cells.

Pathobiology of breast cancer, screening, and identifying new treatments are usually investigated by using Breast cancer cell lines [9, 10]. Cell lines offer several advantages such as the ease of pharmacological and genetic manipulation, the availability of diverse functional assays, and, in some research, the pure cancerous epithelial population (without contamination from stromal cells). MCF7 cell line is one of the breast cancer models that has progesterone and estrogen receptors but lacks ERBB2/HER2 receptors. On the other hand, SKBR3 cell line is used as a model that has progesterone and estrogen receptors but lacks ERBB2/HER2 receptors. MD468 cell line is also used as a model that lacks all three receptors [11]. Various studies have shown that different lines of breast cancer cells, especially the MD468 cell line, are resistant to different treatment methods [12]. Therefore, it is necessary to investigate different signaling pathways along with determining their role in these cell lines in order to find an effective treatment for all types of breast cancer.

In this study, our results showed that the expression of *LDLR* and *LRP6* genes is significantly increased in MCF7 and MD468 cells compared to SKBR3 cells. Similarly, *LDLR* expression is elevated in breast tumor tissues and the accumulation of cholesteryl ester is linked to higher Ki67 expression and unfavorable clinical outcome [13]. Breast cancer cells with high expressions of *LRP1* and *LDLR* take up a greater amount of LDL-C from the bloodstream [14]. The uptake of cholesterol by tumor cells can be transformed into 27-hydroxycholesterol, which, similar to estrogen, might contribute to the epithelial-to-mesenchymal transition process in breast cancer cells that possess estrogen receptors [15, 16]. LRP6, which functions as a receptor for the Wnt/β-catenin pathway, plays a direct role in the development of breast tumors. Consistent with our results, L et al reported that Wnt ligands and LRP6 receptor are overexpressed in triple negative breast cancer cells [17]. Suppressing or deactivating the *LRP6* gene in SUM1315 cells results in the restoration of epithelial markers and decreased abilities for self-renewal and metastasis [18].

In conclusion, all in all, these findings suggest that by using more advanced multicellular and 3D tumor-based systems with patient-derived cells, a deeper understanding of the function of LRP6 and LDLR in breast cancer progression can be found. Therefore, they will be potential therapeutic targets in the future.

### Acknowledgment:

This study is financially supported by Shiraz University of Medical Sciences Grant No. 10700.

### Conflict of Interest:

The authors report no conflicts of interest. 

### Authors’ Contribution:

PM conceived and designed the analysis, verified the analytical methods, monitored the data of this study. HB and ME contributed in data collection, analysis, interpretation in the literature search and drafted the manuscript.
